# An exploratory study of altered regional homogeneity in Parkinson’s disease with depression

**DOI:** 10.3389/fpsyt.2026.1771679

**Published:** 2026-03-05

**Authors:** Shihua Liu, Xudong Zhu, Yan Chen, Chao Zhang, Xiaowei Zhu, Rumeng Zhang, Lei Chen, Bin Li, Ping Zhong

**Affiliations:** 1 Department of Neurology, Suzhou Hospital of Anhui Medical University, Suzhou, Anhui, China; 2 Department of Radiology, Suzhou Hospital of Anhui Medical University, Suzhou, Anhui, China

**Keywords:** depression, neuroimaging biomarkers, Parkinson’s disease, regional homogeneity, resting-state functional MRI

## Abstract

**Background:**

Depression is a prevalent non-motor symptom in Parkinson’s disease (PD), yet its pathogenesis is unclear and biomarkers are lacking. This rs-fMRI study used Regional Homogeneity (ReHo) to explore neural correlates in PD with depression (DPD).

**Methods:**

We included 23 DPD, 24 non-depressed PD (NDPD), and 20 healthy controls (HC). ReHo analysis was applied to identify regional brain activity differences. Correlations between ReHo values and depression severity (HAMD scores) were examined. ROC analysis assessed the diagnostic utility of ReHo changes.

**Results:**

Compared to NDPD, DPD showed increased ReHo in the left inferior temporal gyrus (ITG) and decreased ReHo in the right middle frontal gyrus (MFG), left insula, and left hippocampus. ReHo in left ITG positively correlated with HAMD scores (*r* = 0.4347, *P* = 0.0023), while right MFG (*r* = -0.5262, *P* = 0.0001), left insula, and left hippocampus (*r* = -0.4049, *P* = 0.0048) showed negative correlations. ROC analysis indicated that ReHo in the left insula and hippocampus could distinguish DPD (AUC = 0.8062).

**Conclusion:**

DPD is associated with distinct ReHo alterations. Abnormalities in the left ITG, right MFG, left insula, and left hippocampus may reflect the neural basis of DPD. Our exploratory analyses suggest that altered ReHo in the left insula and left hippocampus may hold potential as neuroimaging biomarkers.

## 1 Introduction

Parkinson’s disease (PD) represents the second most common neurodegenerative disorder, characterized clinically by cardinal motor symptoms-bradykinesia, resting tremor, and rigidity-alongside non-motor symptoms (NMS) including sensory disturbances, affective disorders, and sleep disturbances (1). Crucially, NMS, particularly depression, profoundly impair the quality of life in PD patients. Epidemiological studies indicate that approximately 20%-40% of PD patients exhibit comorbid depressive symptoms, a prevalence significantly higher than that in age-matched healthy populations (2–4). Nevertheless, only 26% receive targeted treatment, while 20%-60% of cases remain undiagnosed or untreated (3). Parkinson’s disease with depression (DPD) not only accelerates cognitive decline and motor deterioration but also correlates with increased disability rates and healthcare burdens (4). However, the pathophysiological mechanisms underlying DPD remain elusive, and clinical diagnosis still relies on subjective scale-based assessments, lacking objective biological markers (5). This critical gap severely hampers the development of early interventions and precision therapeutic strategies.

In recent years, resting-state functional magnetic resonance imaging (rs-fMRI) technology has provided a novel approach for investigating the neural mechanisms underlying DPD (6–8). Rs-fMRI detects blood-oxygen-level-dependent (BOLD) signals to reflect spontaneous neuronal activity, serving as a method to investigate intrinsic brain function during rest. Unlike task-based fMRI, rs-fMRI requires no specific cognitive tasks, instead recording neural activity while subjects remain awake and calm. This technique has gained increasing traction in PD research (9). Regional Homogeneity (ReHo), an emerging rs-fMRI analytic approach, quantifies regional voxel-wise synchronization using Kendall’s coefficient of concordance (KCC, or Kendall’s W). This metric evaluates the coherence of spontaneous activity within localized brain areas (10). ReHo has been extensively applied in neuropsychiatric disorders, including major depressive disorder (11), schizophrenia (12), Alzheimer’s disease (13), epilepsy (14), and Parkinson’s disease (15, 16).

The pathogenesis of DPD remains elusive. Consequently, rs-fMRI-based biomarkers for DPD have become a major research focus in recent years. Previous studies have reported the following findings: widespread weakened connectivity between the temporo-occipital visual cortex and the prefrontal-limbic network in DPD patients (17); the degree of reduced functional connectivity within the medial geniculate network correlates with the severity of depression in DPD patients (7); cortical gyrification may serve as a potential neuroimaging marker for depression severity in PD patients (18); and reduced volumes of the amygdala and hippocampal subfields are associated with the severity of depressive symptoms (19). However, Whether ReHo alterations in DPD-associated depression exhibit disease-specific patterns remains debated, with existing evidence largely derived from small samples or studies inadequately controlling for motor symptom confounds; The distinctive ReHo signatures in DPD have not been systematically characterized; The correlation between ReHo abnormalities and depression severity in DPD remains poorly elucidated (17, 20).

This study aims to employ rs-fMRI using ReHo analysis, in conjunction with strictly matched cohorts of DPD and non-depressed PD (NDPD) patients, to investigate: 1) Whether there exist characteristic brain regions exhibiting altered ReHo in DPD; 2) Whether ReHo values in these differential brain regions correlate with the severity of depression in PD patients; 3) The diagnostic efficacy of ReHo value changes in specific brain regions for DPD. The study aims to provide imaging evidence for the pathophysiological mechanisms underlying DPD and to develop an objective ReHo-based diagnostic biomarker.

## 2 Materials and methods

### 2.1 Participants

This study enrolled 23 patients with DPD recruited between January 2023 and December 2024, selecting 24 gender- and age-matched patients with NDPD and 20 healthy controls (HC). All patients with DPD and NDPD had clinically confirmed primary PD, met the 2015 Movement Disorder Society (MDS) clinical diagnostic criteria for PD (1). Diagnosis of DPD additionally required fulfillment of the Diagnostic and Statistical Manual of Mental Disorders, Fifth Edition (DSM-V) criteria in the context of established PD and a score of ≥14 on the 17-item Hamilton Depression Rating Scale (HAMD-17). All diagnoses were established with the participation of at least two neurologists specializing in movement disorders.

Inclusion criteria for PD patients: 1) No antidepressants/anxiolytics within 2 months; 2) Stable anti-parkinsonian medication regimen >28 days; 3) Right-handedness; 4) Ability to complete assessments independently or with caregiver assistance. Exclusion criteria: 1) Parkinson-plus syndromes (e.g., multiple system atrophy, progressive supranuclear palsy, dementia with Lewy bodies) or secondary parkinsonism (e.g., vascular/drug-induced parkinsonism); 2) Severe psychiatric disorders (e.g., schizophrenia); 3) Major systemic diseases (respiratory/cardiovascular/digestive); 4) Severe cognitive impairment precluding cooperation. HC inclusion criteria: 1) Absence of psychiatric/cognitive disorders; 2) No major systemic diseases; 3) Right-handedness; 4) No structural abnormalities on brain MRI.

This study was approved by the Ethics Committee of Suzhou Hospital of Anhui Medical University (Approval No. A2023026). Written informed consent was obtained from all participants.

### 2.2 Clinical characteristic measurement

Demographic and clinical information was collected for all PD patients and control subjects. Motor severity and disease severity were assessed using the Unified Parkinson’s Disease Rating Scale Part III (UPDRS-III) and the Hoehn & Yahr (H&Y) stage. The severity of depression in PD patients was quantified using HAMD-17. Patients with HAMD-17 scores ≥ 14 points were defined as the DPD group, while those with scores < 14 points were defined as the NDPD group.

### 2.3 Image data acquisition

Prior to neuroimaging, all participants underwent a >12-hour withdrawal from oral antiparkinsonian agents. Scanning was performed on a Philips Ingenia 3T MRI system equipped with a standard head coil. Subjects were positioned supine with head immobilization using foam padding. During acquisition, participants were instructed to maintain rest with eyes closed, avoiding intentional cognitive or motor activities. Structural imaging: High-resolution T1-weighted volumes were acquired via 3D T1W-TFE sequence (Parameters: repetition time (TR) = 6.6 ms, echo time (TE) = 3 ms, flip angle (FA) = 12°, number of slices = 170, slice thickness = 1 mm, slice gap = 1 mm, field of view (FOV) = 240 × 240 mm², matrix size = 512 × 512, and voxel size = 0.5 × 0.5 × 1 mm³). Functional imaging: Resting-state fMRI data were obtained using gradient-echo EPI (8-minute duration; Parameters: TR = 2000 ms, TE = 30 ms, FA = 90°, number of slices = 33, slice thickness = 3.5 mm, slice gap = 0.7 mm, FOV = 224 × 224 mm², matrix size = 128 × 128, and voxel size = 1.75 × 1.75 × 4.2 mm³).

### 2.4 Data processing and ReHo index calculation

Rs-fMRI data were preprocessed using REST plus v1.27 (http://www.restfmri.net/forum/restplus). The preprocessing pipeline comprised: 1) Initial volume removal: Exclusion of the first 10 time points to achieve longitudinal magnetization equilibrium and mitigate scanner acclimatization effects. 2) Slice-timing correction: Temporal realignment for inter-slice acquisition delay compensation. 3) Motion correction: Rigid-body realignment to the first volume, with subjects excluded if exhibiting >3 mm maximum translation or >3° rotation. 4) Spatial normalization: Coregistration to T1-weighted structural images, followed by tissue segmentation and nonlinear warping to the Montreal Neurological Institute (MNI) template via deformation fields. 5) Linear detrending: Elimination of signal trends associated with scanner drift artifacts. 6) Nuisance regression: Incorporation of covariates including Friston-24 motion parameters, cerebrospinal fluid (CSF), and white matter signals. 7) Bandpass filtering: Frequency-based noise reduction (0.01-0.08 Hz) to suppress low-frequency drifts and physiological high-frequency noise.

ReHo computation was performed as follows: 1) Kendall’s Concordance Calculation: The ReHo value for each voxel was derived by computing KCC between its time series and those of its 26 nearest neighboring voxels. For standardization, individual voxel-wise ReHo values were normalized by dividing by the global mean ReHo across the whole brain. 2) Spatial Smoothing: The normalized ReHo maps were smoothed using a Gaussian kernel with a full-width at half-maximum (FWHM) of 4 mm.

### 2.5 Statistical analysis

Demographic and clinical data were analyzed using GraphPad Prism v9.0 (GraphPad Software, USA) across DPD, NDPD, and HC groups. Intergroup comparisons were performed as follows: One-way analysis of variance (ANOVA) for continuous variables among three groups; Pearson’s chi-square test (χ²) for categorical variables (sex); Continuous variables including age, H &Y stage, UPDRS-III scores, HAMD-17 scores, and levodopa equivalent daily dose (LEDD) were analyzed using independent samples t-test.

Neuroimaging analysis: ReHo statistical maps underwent analysis of covariance (ANCOVA) with *post-hoc* testing in REST 1.8 toolkit. Statistical significance was defined at a voxel-level threshold of *P* < 0.01 and cluster-level threshold of *P* < 0.05 (family-wise error corrected). Significant clusters were overlaid onto the standard CH2 template. Automated Anatomical Labeling (AAL) atlas identified anatomical labels of differential brain regions, with Montreal Neurological Institute (MNI) coordinates, cluster size (voxels), and peak t-values recorded.

Correlational analysis and statistical validation: A mask file was created based on the statistically significant brain regions identified from the ANOVA-corrected results. Subsequently, the mean ReHo value within each mask was extracted, yielding one average ReHo value per subject for each significant cluster. For all patients in both the DPD and NDPD groups, the mean ReHo value of each significant cluster was included in a Pearson correlation analysis with the corresponding HAMD-17 score. Gaussian random field (GRF) theory correction addressed multiple comparisons (voxel *P* < 0.05, cluster *P* < 0.05, two-tailed).To evaluate the discriminative power between DPD and NDPD groups, receiver operating characteristic (ROC) curve analysis was employed, with diagnostic accuracy quantified by the area under the curve (AUC). Statistical significance was defined as *P* < 0.05 (two-tailed).

## 3 Results

### 3.1 Demographic and clinical characteristics

No statistically significant differences were observed in gender distribution or age among the DPD, NDPD, and HC groups. The DPD and NDPD groups demonstrated comparable scores on the UPDRS-III, H&Y staging, and LEDD. The DPD group exhibited significantly higher HAMD-17 scores compared to both the NDPD and HC groups (**Table 1**).

**Table 1:** Comparison of demographic and clinical characteristics across groups ( $\bar{x}$ ± *s*).

Demographic and clinical data	DPD group (*n* = 23)	NDPD group (*n* = 24)	HC (*n* = 20)	*F*/*χ²*/*t*	*P* value
Age/year	65.78 ± 9.06	63.67 ± 8.38	62.70 ± 6.57	0.822^a^	0.444
Sex, male/female	12/11	12/12	10/10	0.029^b^	0.986
UPDRS-III score	35.39 ± 12.04	33.38 ± 10.33	NA	0.704^c^	0.485
H &Y stage	2.59 ± 0.96	2.25 ± 0.72	NA	1.354^c^	0.183
HAMD-17 scores	28.00 ± 6.69	14.17 ± 3.12	NA	30.83^c^	<0.001
LEDD, mg/d	532.6 ± 152.9	500.0 ± 153.1	NA	0.730^c^	0.469

UPDRS-III, the Unified Parkinson’s Disease Rating Scale Part III; H &Y stage: the Hoehn & Yahr stage; HAMD-17: 17-item Hamilton.

Depression Rating Scale; LEDD, levodopa equivalent daily dose. a: one-way ANOVA test; b: χ2 test; c: Two independent samples *t* test.

### 3.2 ReHo differences in brain regions

ANCOVA revealed significant inter-group differences in ReHo primarily localized to the left inferior temporal gyrus (ITG), right middle frontal gyrus (MFG), left insula and left hippocampus (**Table 2**).

**Table 2:** Brain regions with significant differences in ReHo among the three groups.

Brain regions (AAL)	Cluster size	Peak MNI coordinates			*F* value
		X	Y	Z	
Left Inferior Temporal Gyrus	250	-39	-9	-36	10.0801
Right Middle Frontal Gyrus	429	39	54	12	10.4113
Left Insula/Left Hippocampus	262	-39	-12	-3	10.5336

AAL, Automated Anatomical Labeling; MNI, Montreal Neurological Institute.

**Figure 1:**
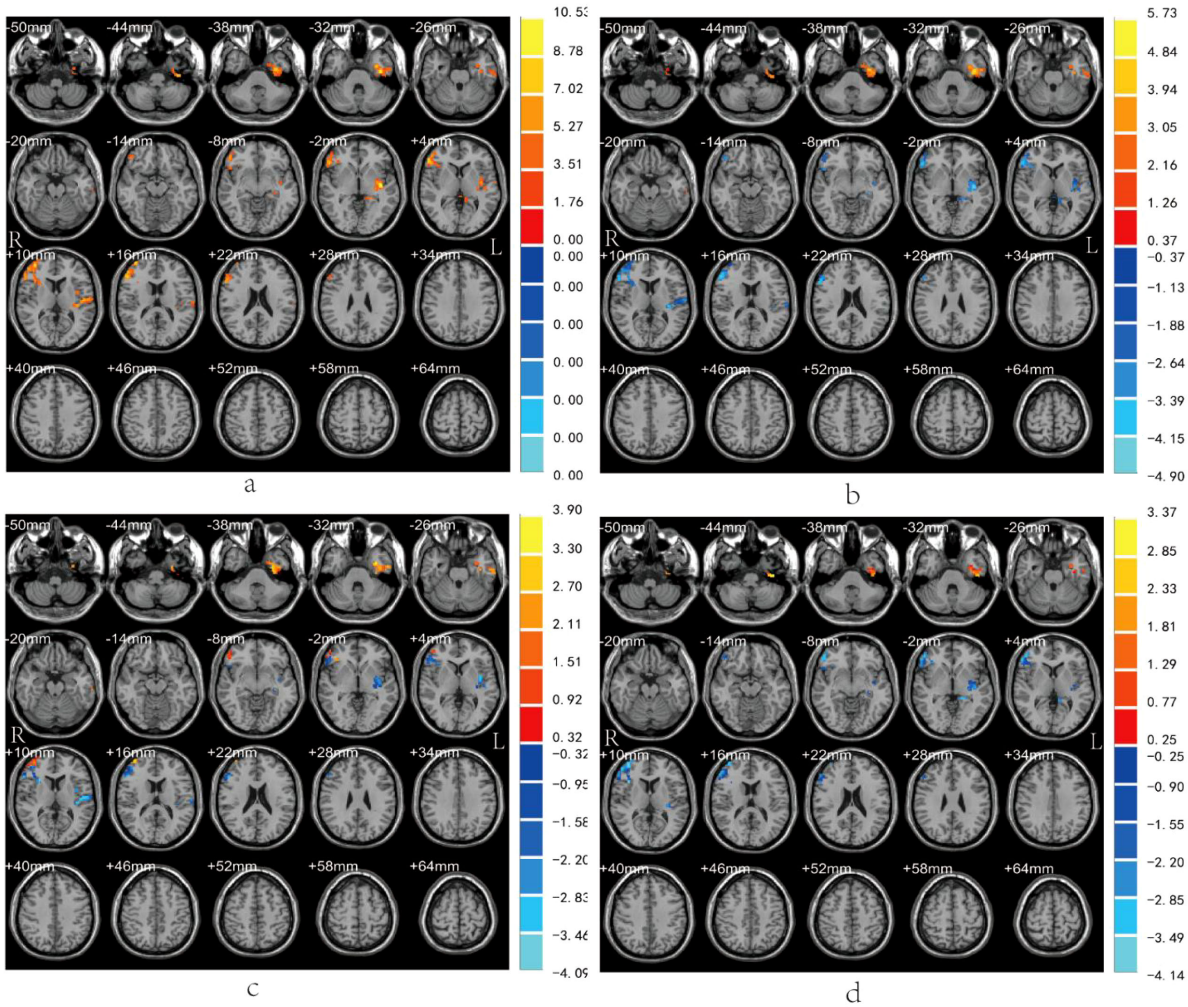
Brain regions with significant ReHo differences. **(a)** Brain regions showing significant ReHo differences among the three groups (red regions). **(b)** Between-group differences between the DPD and HC groups. The red region indicates the left inferior temporal gyrus (ITG) where ReHo was significantly higher in the DPD group than in the HC group (DPD > HC). The blue regions indicate the right middle frontal gyrus (MFG), left insula, and left hippocampus, where ReHo was significantly lower in the DPD group than in the HC group (DPD < HC). **(c)** Between-group differences between the NDPD and HC groups. The red regions indicate the left ITG and right precuneus where ReHo was significantly higher in the NDPD group than in the HC group (NDPD > HC). The blue regions indicate the right MFG and left insula where ReHo was significantly lower in the NDPD group than in the HC group (NDPD < HC). **(d)** Between-group differences between the DPD and NDPD groups. The red region indicates the left ITG where ReHo was significantly higher in the DPD group than in the NDPD group (DPD > NDPD). The blue regions indicate the right MFG, left insula, and left hippocampus where ReHo was significantly lower in the DPD group than in the NDPD group (DPD < NDPD). The color bar in **(a)** represents the *F*-values from the ANCOVA analysis among the three groups, whereas the color bars in **(b)**, **(c)**, and **(d)** represent the *t*-values from the *post hoc* tests. Red regions indicate brain areas with significantly increased ReHo, and blue regions indicate the opposite (multiple comparisons were corrected using Gaussian random field correction, voxel-level *P* < 0.01, cluster-level *P* < 0.05). R, right; L, left.

**Figure 2:**
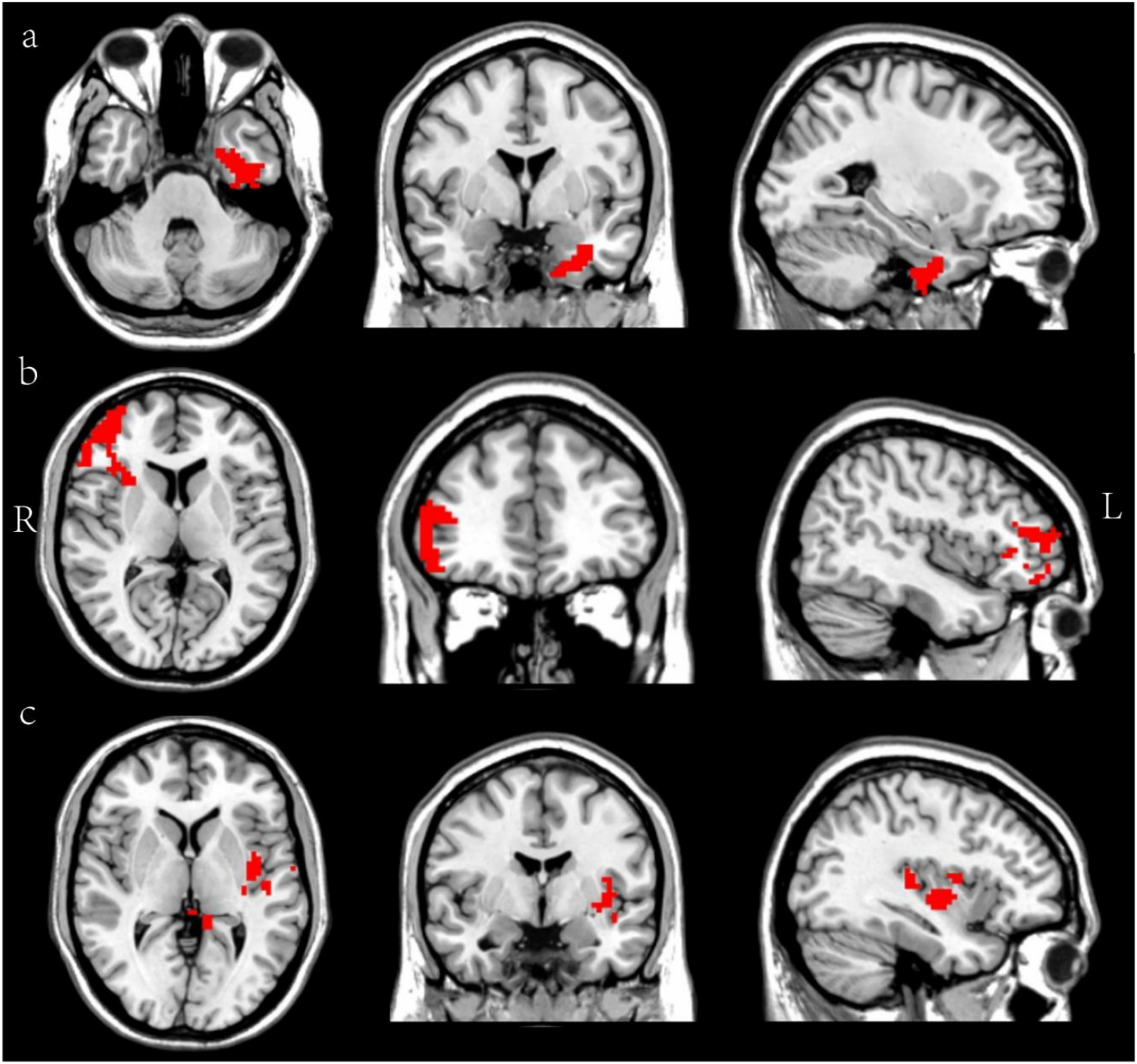
Displays orthogonal views of brain regions with ReHo differences **(a–c)** show orthogonal views (coronal, sagittal, and axial) to clearly illustrate the brain regions exhibiting ReHo differences between the DPD and NDPD groups. The differential brain region in panel a is the left inferior temporal gyrus (ITG), in panel b is the right middle frontal gyrus (MFG), and in panel c are the left insula and left hippocampus. The red color indicates only that a ReHo difference exists in that region. R, right; L, left.

**Figure 3:**
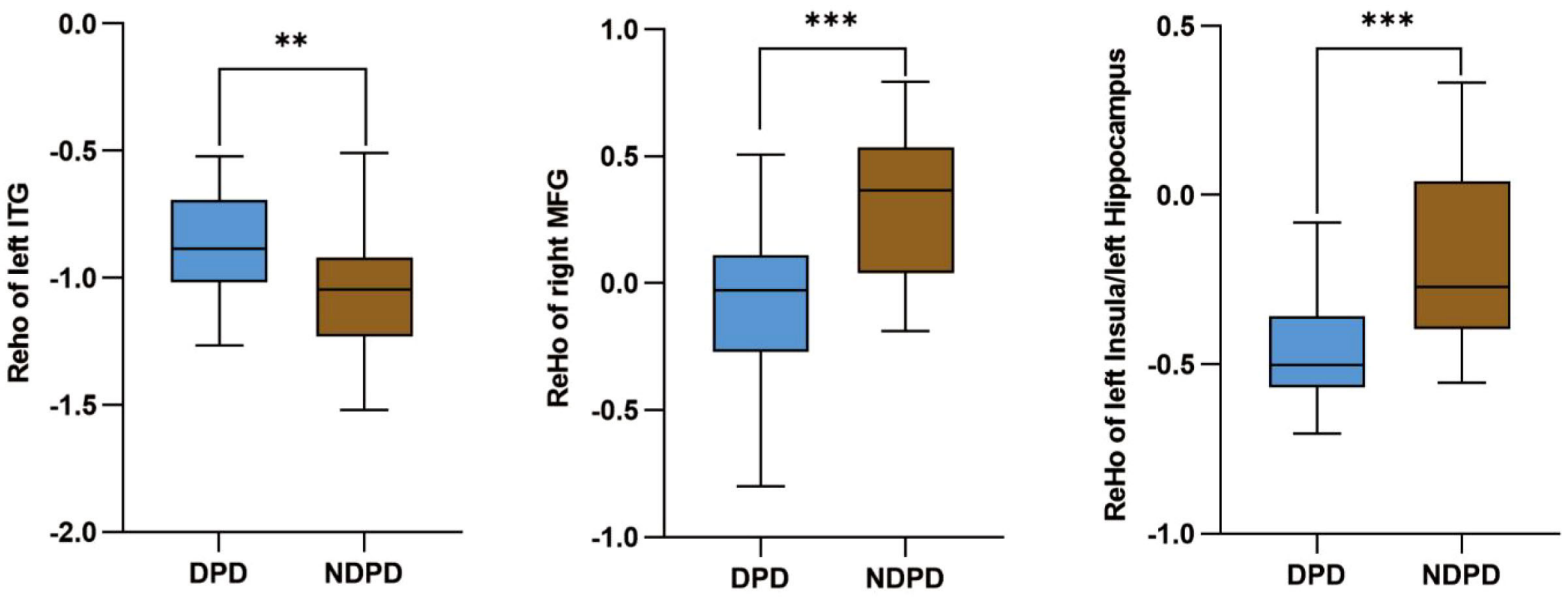
Comparison of ReHo values in brain regions with significant differences between the DPD and NDPD groups. The DPD group exhibited significantly higher ReHo values in the left inferior temporal gyrus (ITG), and significantly lower ReHo values in the right middle frontal gyrus (MFG), left insula, and left hippocampus compared to the NDPD group (all *P* < 0.05). "**" indicates *P* < 0.01, and "***" indicates *P* < 0.001.

*Post-hoc* tests demonstrated that compared with the HC group: The DPD group exhibited significantly increased ReHo in the left ITG, but decreased ReHo in the right MFG, left insula and left hippocampus; the NDPD group showed elevated ReHo in the right precuneus and left ITG, with reduced ReHo in the right MFG, left insula. Furthermore, relative to the NDPD group, the DPD group displayed increased ReHo in the left ITG and decreased ReHo in the right MFG, left insula and left hippocampus (**Table 3**; **Figure 1**). Orthogonal views (coronal, sagittal, axial) of these between-group ReHo differences are presented in **Figure 2**. Consistent with these results, direct comparison confirmed that the DPD group had higher ReHo values in the left ITG and lower ReHo values in the right MFG and the left insula/hippocampus compared to the NDPD group ((*P* < 0.05) (**Figure 3**).

**Table 3:** Brain regions with significant differences in ReHo between groups.

Comparison results and regions (AAL)	Cluster size	Peak MNI coordinates			*t* value
		X	Y	Z	
DPD>HC					
Left Inferior Temporal Gyrus	250	-42	-9	-33	5.7282
DPD<HC					
Right Middle Frontal Gyrus	401	54	30	18	-4.4273
Left Insula/Left Hippocampus	249	-42	-15	-3	-4.9027
NDPD>HC					
Right Precuneus	167	-18	-63	36	4.1622
Left Inferior Temporal Gyrus	186	18	-33	39	3.8351
NDPD<HC					
Right Middle Frontal Gyrus	111	21	-105	3	-4.3867
Left Insula	87	-33	-99	3	-4.5374
DPD>NDPD					
Left Inferior Temporal Gyrus	148	-39	-18	-45	3.3718
DPD< NDPD					
Right Middle Frontal Gyrus	396	39	54	12	-4.1428
Left Insula/Left Hippocampus	135	-27	-27	-12	-3.2201

AAL, Automated Anatomical Labeling; MNI, Montreal Neurological Institute.

### 3.3 Correlation analysis between altered ReHo values and HAMD scores

**Figure 4:**
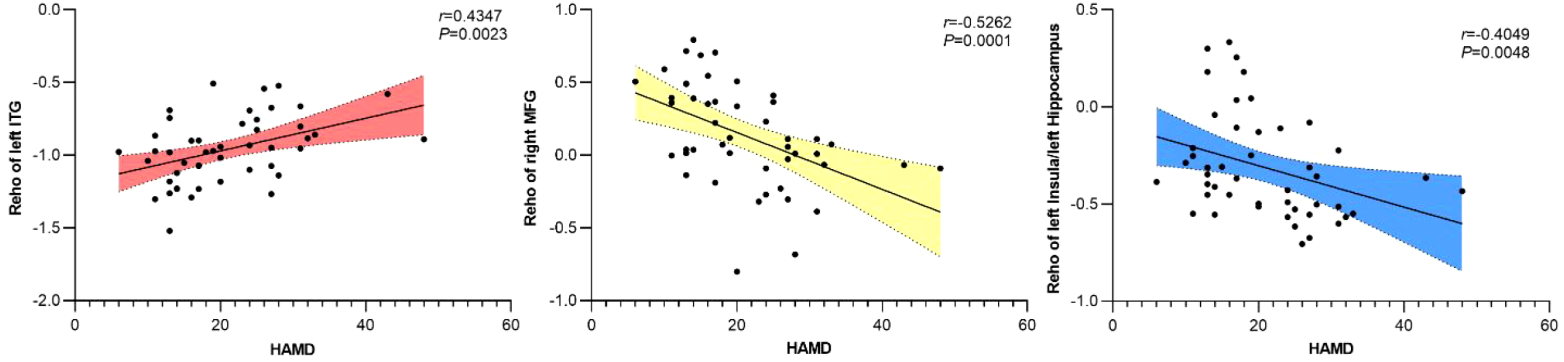
Correlation analysis between ReHo values in brain regions with significant differences and HAMD scores in PD patients. ReHo values in the left inferior temporal gyrus (ITG) showed a significant positive correlation with HAMD scores in PD patients (*r* = 0.4347, *P* = 0.0023). In contrast, ReHo values in the right middle frontal gyrus (MFG), left insula, and left hippocampus exhibited significant negative correlations with HAMD scores (*r* = -0.5262, *P* = 0.0001; and *r* = -0.4049, *P* = 0.0048, respectively).

Correlation analysis was performed between the ReHo values of brain regions showing significant differences between the DPD and NDPD groups and their HAMD scores. The results revealed a significant positive correlation between ReHo values in the left ITG and HAMD scores (*r* = 0.4347, *P* = 0.0023). Conversely, significant negative correlations were observed between ReHo values in the right MFG and HAMD scores (*r* = -0.5262, *P* = 0.0001), as well as between ReHo values in the left insula/hippocampus and HAMD scores (*r* = -0.4049, *P* = 0.0048) (**Figure 4**).

### 3.4 Diagnostic performance of ReHo values in discriminating DPD

**Figure 5:**
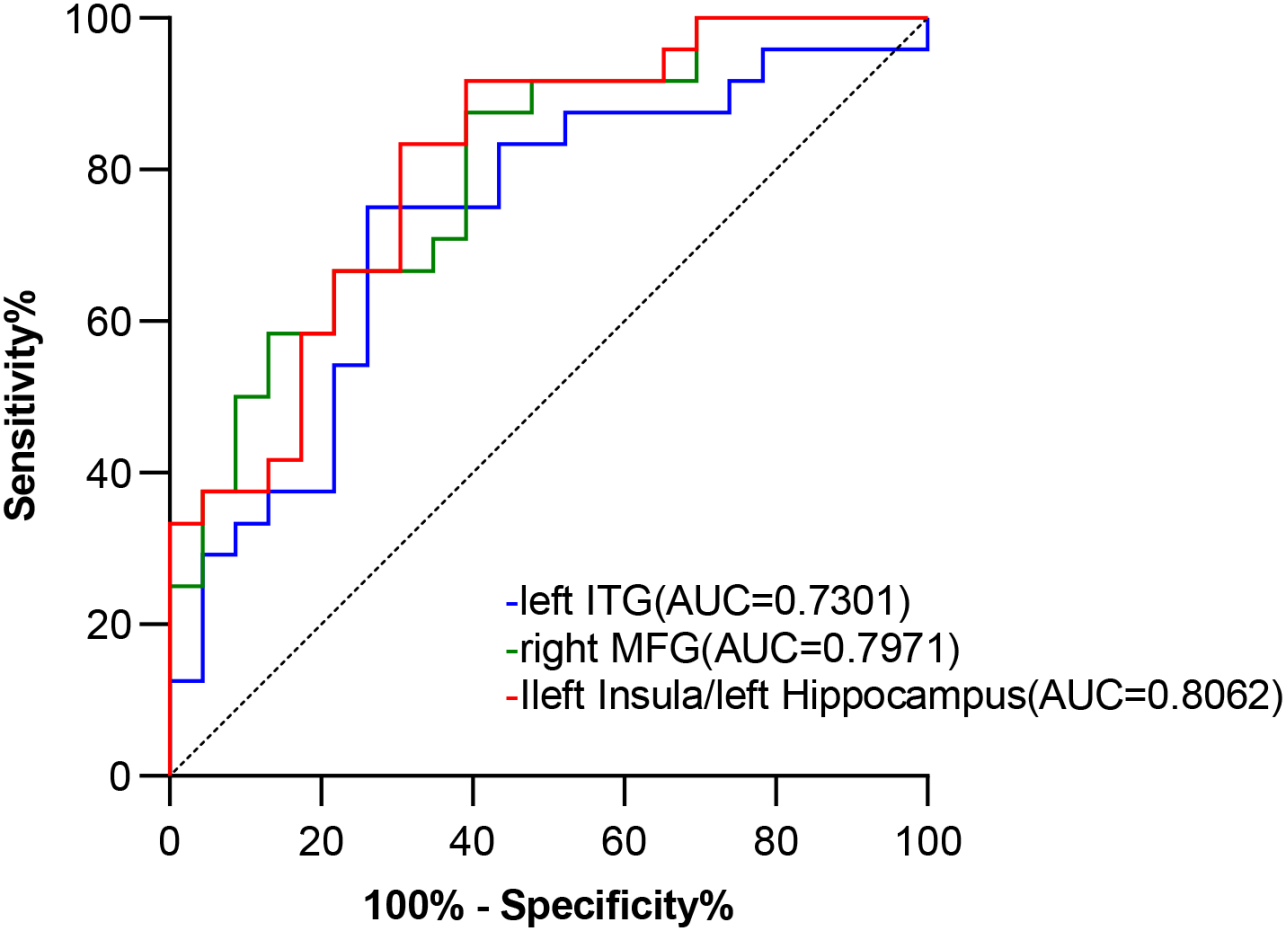
Diagnostic value of ReHo values in brain regions with significant differences for discriminating DPD ROC curve analysis evaluated the efficacy of ReHo alterations in differentiating DPD. The results showed that the area under the curve (AUC) was 0.7301 for the left inferior temporal gyrus (ITG), 0.7971 for the right middle frontal gyrus (MFG), and 0.8062 (95% confidence interval: 0.683 to 0.930, *P* < 0.001) for the left insula and left hippocampus.

The brain regions exhibiting significant ReHo differences between the DPD and NDPD groups were the left ITG, the right MFG, and the left insula/left hippocampus. The ReHo values from these significantly different brain regions were employed to evaluate their discriminative efficacy in distinguishing between the two groups. ROC curve analysis was employed to evaluate the ability of altered ReHo values in the identified differential brain regions to distinguish DPD. The results demonstrated that the AUC for the left ITG was 0.7301. The AUC for the right MFG was 0.7971. The AUC for the left insula/left hippocampus was 0.8062 (95% *CI*: 0.683–0.930, *P* < 0.001), as shown in **Figure 5**.

## 4 Discussion

This study employed rs-fMRI with ReHo analysis to investigate the characteristics of brain functional activity in patients with DPD. The results demonstrated significant ReHo alterations in DPD patients within brain regions including the left ITG, right MFG, left insula, and left hippocampus. These alterations were significantly correlated with the severity of depressive symptoms in DPD. Furthermore, ReHo changes in the left insula and left hippocampus exhibited high discriminative power for diagnosing DPD (AUC = 0.8062), suggesting their potential as neuroimaging biomarkers for DPD.

This study investigated the alterations in ReHo among DPD, NDPD, and HC, revealing complex patterns of cerebral functional changes associated with PD and comorbid depression. A key finding is that alterations in certain brain regions were observed in both NDPD and DPD groups compared to HC, such as increased ReHo in the left ITG and decreased ReHo in the right MFG. This suggests that these changes may reflect core pathophysiological processes of PD itself, rather than being specific markers of depression (21). When interpreting DPD-specific alterations, we clearly distinguished the reference baselines: changes in DPD relative to HC (e.g., reduced ReHo in the left insula/hippocampal regions) may represent the combined or interactive effects of PD and depression; whereas changes in DPD relative to NDPD (e.g., further reduction of ReHo in the right MFG in DPD) are more likely to directly reflect the contribution of the depressive dimension. Of particular interest is the alteration pattern in the right MFG. This study observed that the NDPD group already showed a decreasing trend in ReHo in this region compared to HC (NDPD < HC), and this reduction became more pronounced in the DPD group (DPD < HC and DPD < NDPD). This pattern supports a potential “compensation-decompensation” neural mechanism hypothesis (22). In the early stages of PD or in the absence of depression, other brain networks (e.g., the cognitive control network) may partially compensate for dysfunction in the basal ganglia-thalamocortical circuits by enhancing synchronization-a compensatory effort-to maintain normal emotional and cognitive functions (23). However, as the disease progresses or depression develops, this compensatory mechanism may reach its limit or fail, leading to a significant decrease in ReHo in key nodes such as the right MFG. The functional deactivation of this region may be directly associated with the emergence of depressive symptoms, such as impaired executive function and negative emotional bias (24). On the other hand, brain regions that showed specific alterations across multiple comparisons (DPD < HC, DPD < NDPD), such as the left insula and hippocampus, are more likely to be specific neural markers of depression comorbid with PD. These regions are involved in interoceptive awareness, emotional integration, and memory processing, and their dysfunction is highly relevant to the pathophysiology of depression (25). Our findings suggest that within the context of PD, reduced ReHo in these limbic and paralimbic regions may represent an independent contributing factor to the overlay of depressive symptoms.

### 4.1 Neural mechanisms of ReHo alterations in DPD patients

#### 4.1.1 Increased ReHo in the left ITG and impaired emotion regulation

This study revealed a significant increase in ReHo values within the left ITG in DPD patients compared to NDPD. The left ITG, belonging to the higher-order association cortex, is implicated in emotion processing, semantic memory, and social cognition. Hyperactivation within temporal lobe cortices, potentially reflecting aberrantly enhanced processing of negative emotions, is frequently observed in patients with major depressive disorder (26, 27). In PD patients, degeneration of the dopaminergic neurotransmitter system may disrupt functional connectivity within limbic circuits (e.g., the amygdala-temporal lobe circuit), contributing to impaired emotion regulation (28). Existing research has linked metabolic abnormalities in this region to negative emotional biases in depression (29–31). The elevated ReHo observed here suggests a heightened processing bias toward negative emotional stimuli in DPD patients. This finding aligns with previous reports of hyperactivation in the posterior default mode network (DMN) in individuals with depressive disorders (8). Our results support the view that increased ReHo in the left inferior temporal gyrus of DPD patients may represent aberrant neural compensation for depressive symptomatology, analogous to the pattern of temporal lobe hyperactivation observed in primary depression (32).

#### 4.1.2 Decreased ReHo in the right MFG and impairments in emotion and cognitive control

This study demonstrated a significant decrease in ReHo values within the right MFG in DPD patients. As a key component of the dorsolateral prefrontal cortex (DLPFC), the MFG plays a crucial role in emotion regulation and cognitive control (33). The observed reduction in its functional activity (as indicated by lower ReHo) may reflect an impairment in top-down emotion regulation in DPD patients. This finding aligns with the clinical characteristics of diminished executive control function commonly observed in individuals with depression (34, 35). Depressive symptoms in PD patients are frequently accompanied by “executive dysfunction” and “negative cognitive bias”, manifesting as difficulties in suppressing negative thoughts and modulating emotional responses (36). Our results suggest that the decreased ReHo in the right MFG may reflect functional impairment within the prefrontal-striatal circuit in DPD patients, leading to a diminished capacity to regulate emotional information. This discovery is consistent with previous studies on PD-related depression (37–39), supporting the view that dysfunction within the prefrontal-limbic system represents one of the core neural mechanisms underlying DPD (7).

#### 4.1.3 Reduced ReHo in the left insula and left hippocampus related to emotion-somatic integration impairment

Another key finding of this study is the significantly reduced Regional Homogeneity (ReHo) values in the left insula and left hippocampus of patients with depression in Parkinson’s disease (DPD). The insula, a critical hub of the Salience Network (SN), is responsible for integrating interoceptive signals (such as emotion, pain, and autonomic responses) and directing attentional resource allocation (7). In patients with major depressive disorder (MDD), insular dysfunction is closely associated with emotional blunting and somatic symptoms (40). Altered insular function in PD patients may involve dual dysregulation of the dopaminergic and serotonergic (5-HT) systems. Animal models demonstrate that PD-related substantia nigra degeneration can impact synaptic plasticity within the insula, while reductions in 5-HT neurotransmission may further exacerbate impairments in emotional perception (41). Our findings support the hypothesis that reduced ReHo in the left insula of DPD patients may reflect impaired emotion-somatic integration function, potentially leading to the exacerbation of depressive symptoms such as anhedonia and fatigue. Notably, the reduced ReHo value in the left hippocampal region likely carries dual pathological significance. On the one hand, as a core structure of the limbic system, hippocampal hypofunction may directly contribute to the formation of depression-related affective disturbances (19). On the other hand, considering the inherent neurodegenerative nature of PD, abnormalities in this region may simultaneously reflect the superimposed effects of damage to both dopaminergic and non-dopaminergic systems during disease progression (42).

### 4.2 Correlation between altered ReHo in differential brain regions and depression severity in PD patients

Another significant finding of this study is the significant correlation between ReHo values in these differential brain regions and HAMD scores in PD patients. A significant positive correlation was observed between ReHo values in the left ITG and HAMD scores in PD patients. This suggests that hyper-synchronization in this region may directly contribute to the neuropathological processes underlying depressive symptoms, consistent with previous research findings (43). Conversely, ReHo values in the right MFG, left insula, and left hippocampus showed significant negative correlations with HAMD scores. The functional connectivity between the ventral visual pathway (including the ITG) and medial temporal lobe structures (such as the hippocampus) plays a crucial role in emotional memory and visual-emotional integration (44, 45). Abnormalities in the structure and functional connectivity of the hippocampus are associated with the severity of DPD (19, 42). This pathway exhibits altered connectivity in patients with depression (46), which may explain the link between visual-emotional processing abnormalities and depressive symptoms. Furthermore, the insula, as a hub of the salience network, is tightly connected to the temporal lobe and limbic system via white matter tracts such as the uncinate fasciculus. Alterations in its structural and functional connectivity may affect the integration of emotion, interoception, and cognitive processes, thereby contributing to the neural mechanisms of depression (47, 48). This finding further supports the critical role of prefrontal-limbic neural circuitry imbalance in the development and progression of DPD (49). Clinical Significance of Left Insula and Left Hippocampus ReHo as Potential Biomarkers for DPD.

Currently, the diagnosis of DPD primarily relies on clinical interviews and rating scales (e.g., HAMD), lacking objective biological markers. Through ROC analysis, this study demonstrated that alterations in ReHo within the left insula and left hippocampus possess moderate to high discriminatory power for identifying DPD (AUC>0.8), suggesting their potential utility as adjunctive diagnostic tools. The abnormal ReHo pattern identified provides a potential neuroimaging biomarker for the early detection of DPD. This finding holds significant clinical value: 1) Improved Diagnostic Accuracy: Depressive symptoms in some PD patients may be obscured by motor manifestations (e.g., hypomimia, bradykinesia), potentially leading to misdiagnosis. Objective measurement of left insula and left hippocampus ReHo could aid in distinguishing DPD from NDPD. 2) Guiding Personalized Treatment: If future research confirms that functional alterations in the insula and hippocampus correlate with the efficacy of specific antidepressants (e.g., SSRIs), ReHo analysis could potentially be used to predict treatment response.

In recent years, neuromodulation techniques, such as transcranial magnetic stimulation (TMS) and deep brain stimulation (DBS), have shown promise for treatment-resistant depression (50–52). This study’s finding of key functional abnormalities in the insula and left hippocampus in DPD suggests that targeted neuromodulation of the left insula, hippocampus, or their connected networks may alleviate DPD symptoms.

However, this study has several limitations. First, the cross-sectional design precludes causal inferences regarding the relationship between the observed ReHo alterations and depressive symptoms. Second, the sample size is relatively small, derived from a single center, and the analysis relied on a single neuroimaging metric (ReHo). Third, specific methodological constraints should be noted. The correlational analysis between ReHo in the identified clusters and HAMD scores is descriptive and should not be interpreted as an independent validation due to potential circularity. Similarly, the ROC analysis is exploratory, and the reported diagnostic accuracy (AUC) is likely optimistic in the absence of an independent validation cohort. Future studies employing larger, multi-center cohorts, longitudinal designs, multimodal neuroimaging, and more robust analytical methods (e.g., voxel-wise correlation and training-testing validation) are warranted to confirm these preliminary findings, elucidate their temporal dynamics, and rigorously evaluate their biomarker potential.

## 5 Conclusion

This study employed rs-fMRI using the ReHo method and revealed characteristic ReHo alterations in patients with DPD. Functional changes in the ITG, right MFG, left insula and left hippocampus may underlie the neural mechanisms of DPD, potentially involving the synergistic impairment of networks responsible for emotional processing, cognitive control, and emotion-somatic integration. Our exploratory analyses suggest that altered ReHo in the left insula and left hippocampus may hold potential as neuroimaging biomarkers.

## Data Availability

The original contributions presented in the study are included in the article/supplementary material. Further inquiries can be directed to the corresponding author.
